# Research progress on the intervention of osteoporosis by Chinese herbal monomers based on the Nrf2/NF-κB/MAPK pathway

**DOI:** 10.3389/fendo.2026.1789264

**Published:** 2026-06-24

**Authors:** Wenjie Kou, Chenglong Guo, Xiaomin Lu, Zhe Zhang, Kaiwen Liu, Zhihuan Liu, Bin Jiang, Chengxiang Ma, Jishu Li, Jiandong Gao, Linzhong Cao, Xiaogang Zhang

**Affiliations:** 1 Gansu University of Traditional Chinese Medicine, Lanzhou, Gansu, China; 2 Affiliated Hospital of Gansu University of Traditional Chinese Medicine, Lanzhou, Gansu, China

**Keywords:** bone metabolism, inflammatory response, MAPK pathway, NF-κB pathway, Nrf2 pathway, osteoporosis, oxidative stress, traditional Chinese medicine compounds

## Abstract

Osteoporosis (OP) is a chronic metabolic bone disorder marked by reduced bone mass, damaged microarchitecture, and increased skeletal fragility. Its pathogenesis is complicated, and the central mechanism involves an imbalance between osteoblastic bone formation and osteoclastic bone resorption, together with several pathological processes, including excessive oxidative stress, persistent inflammatory infiltration, and abnormal apoptosis. The Nuclear Factor Erythroid 2-Related Factor 2 (Nrf2), Nuclear Factor-κB (NF-κB), and Mitogen-Activated Protein Kinase (MAPK) pathways are important signaling networks controlling bone metabolism, oxidative stress, and inflammation. These pathways interact through upstream and downstream regulatory molecules. They establish complex regulatory networks through molecular interactions and crosstalk, collectively promoting the initiation and progression of OP. Traditional Chinese medicine (TCM), based on the holistic concept and “syndrome differentiation and treatment,” shows promising clinical value in OP prevention and therapy because of its characteristic multi-target, multi-pathway actions and low toxicity. Extensive pharmacological evidence has confirmed that individual TCM compounds can specifically regulate the Nrf2/NF-κB pathways. By modulating redox homeostasis, inhibiting inflammatory responses, and regulating osteocyte proliferation, differentiation, and apoptosis, these compounds restore bone metabolic equilibrium and produce anti-OP effects. Current reviews mostly focus on individual signaling pathway or single herbal component, lacking systematic sorting of the cross-talk among Nrf2, NF-κB and MAPK networks. This article systematically reviews the major roles of the Nrf2, NF-κB, and MAPK pathways in osteoporosis and discusses the anti-osteoporotic mechanisms of Chinese herbal monomers, thereby providing a theoretical foundation for TCM intervention. Furthermore, it highlights syndrome differentiation and personalized treatment to overcome the limitations of basic research and clarify future clinical translation.

## 1 Introduction

With the rapid aging of the global population, OP has become an important public health issue worldwide, with an increasing incidence among middle-aged and elderly individuals. According to data from the Chinese Guidelines for the Diagnosis and Treatment of OP (2022 Edition), the prevalence of OP among Chinese adults aged ≥50 is 19.2%, with women at 32.1% vs. men at 6.9%. In adults aged ≥65, the prevalence rises to 32.0%, and that in women reaches 51.6% (1). The major risk associated with OP is a fragility fracture, which occurs most frequently at the hip, spine, and wrist. Within one year after hip fracture, cardiovascular events, disability, and mortality rates reach 20%, 50% and 20%–30%. This markedly reduces quality of life and creates a substantial burden on families and healthcare systems. Moreover, OP is not only related to aging. Chronic diseases (diabetes, hyperthyroidism, CKD) and long-term medications (glucocorticoids, antiepileptics) can cause secondary OP, thereby expanding the affected population. Current treatment of OP mainly depends on Western medications: antiresorptive drugs, anabolic agents, and bone metabolism modulators (2). Although bisphosphonates, estrogen therapy, and anabolic agents can improve OP, they are linked to adverse reactions, potential risks, and high costs. Although Western drugs such as RANKL-targeted antibodies are highly specific and show clear efficacy in short-term regulation of bone metabolism, their mechanisms of action are relatively limited. They regulate bone resorption alone and do not fully consider the overall physical condition of the patient. This limits personalized treatment based on pathophysiological differences, and long-term use involves contraindications and possible safety concerns (3). Based on the holistic concept and the system of syndrome differentiation and treatment, TCM can provide individualized treatment according to the specific syndrome pattern of each patient, showing clear complementary advantages in the long-term management of chronic diseases and in improving overall physical constitution. Under the guidance of the holistic concept and syndrome differentiation and treatment, TCM can formulate individualized intervention plans according to different TCM patterns, such as kidney deficiency, spleen deficiency, and blood stasis. In the long-term management of OP, improvement of the overall health status of patients, and implementation of stratified and individualized treatment, TCM has unique clinical advantages that are difficult to achieve with modern Western medicine. This also indicates an important direction for research and application of TCM in the prevention and treatment of OP.

TCM has recognized OP for a long time, classifying it as “bone atrophy,” “bone obstruction” or “consumptive disease”. Its pathogenesis is associated with dysfunction of the kidney, spleen, and liver, with key mechanisms including kidney essence deficiency, spleen-stomach weakness, qi stagnation and blood stasis, and inadequate nourishment of sinews and bones (4). The Huangdi Neijing states: “The kidney governs bones and generates marrow” and “The spleen governs transformation and transportation, being the source of qi and blood,” thereby establishing the central functions of the kidney and spleen in skeletal physiology. Later physicians further proposed that “The liver governs sinews, which attach to bones; sufficient liver blood strengthens sinews and bones,” which refined the TCM theory of OP pathogenesis (5). Accordingly, TCM manages OP by tonifying the kidney and nourishing essence, strengthening the spleen and boosting qi, promoting blood circulation and unblocking collaterals, and strengthening sinews and bones, thereby forming an integrated therapeutic system.

Modern pharmacological studies indicate that TCM produces anti-osteoporotic effects not through a single component or target. Rather, it acts through multiple factors by regulating bone metabolism pathways, oxidative stress, inflammation, and gut microbiota (6). As key components of the bone metabolism regulatory network, the Nrf2, NF-κB, and MAPK pathways show functional abnormalities that constitute an important molecular basis for OP development: impaired Nrf2 pathway activity causes excessive oxidative stress, damages osteoblasts and activates osteoclasts; excessive activation of the NF-κB pathway induces chronic inflammation, aggravates bone resorption and inhibits bone formation; dysregulation of MAPK pathway branches disturbs bone cell proliferation, differentiation, and apoptosis, thereby disrupting bone remodeling equilibrium (7). Importantly, these pathways do not act separately but establish complex crosstalk networks through shared targets, such as oxidative stress and inflammatory mediators, and jointly regulate bone metabolic homeostasis. In recent years, considerable progress has been made in TCM research targeting these pathways for OP. Many TCM monomers (e.g., total flavonoids from Dipsacus asper, polysaccharides from Astragalus membranaceus, diosgenin) have been reported to achieve precise regulation of bone metabolism by modulating key molecules in these pathways. This review summarizes the mechanisms and research progress of TCM monomers targeting the Nrf2/NF-κB/MAPK pathways in OP, providing new insights for prevention and treatment. However, current reviews mainly emphasize individual herbal compounds or single signaling pathways, and a systematic summary of crosstalk within the Nrf2/NF-κB/MAPK network and the multi-target mechanisms of herbal monomers in OP is still lacking. Therefore, this review systematically describes the roles of these three pathways in OP pathogenesis and the regulatory mechanisms of representative herbal monomers. It aims to clarify the common regulatory patterns of different monomer types, fill the gap in systematic reviews of multi-pathway crosstalk, and provide a theoretical basis for developing multi-target anti-osteoporosis drugs and advancing clinical TCM research.

## 2 Biological functions of the Nrf2/NF-κB/MAPK pathway and its mechanism of action in OP

This section summarizes the fundamental functions of the Nrf2, NF-κB, and MAPK pathways, providing a basis for analyzing the mechanisms of TCM monomers. The review mainly focuses on TCM monomers.

### 2.1 Molecular mechanisms of the Nrf2 pathway and its role in OP

Nrf2 is a key transcription factor of the CNC leucine zipper family and is located on human chromosome 2. It contains seven NEH domains with different functions: NEH1: DNA-binding domain, binds ARE; NEH2: Keap1-binding domain, forms a complex with Keap1 through DLG/ETGE motifs; NEH3/4/5: transcriptional activation domains; NEH6: phosphorylation-rich region involved in Nrf2 degradation; NEH7: binds RXRα and suppresses Nrf2 activity (8). Under physiological conditions, Nrf2 forms a complex with Keap1 in the cytoplasm. Keap1 functions as an adaptor, recruiting E3 ubiquitin ligases to promote Nrf2 degradation through the proteasome, thereby maintaining low basal Nrf2 levels (9). Under oxidative stress, drug stimulation, or inflammation, oxidation of Keap1 cysteine residues changes its conformation and weakens Nrf2 binding. Nrf2 then dissociates and translocates into the nucleus (10). After nuclear entry, Nrf2 forms heterodimers with small Maf proteins. These dimers bind to AREs in the promoters of target genes and induce antioxidant, detoxification, and anti-inflammatory genes. These genes include HO-1, GPX, SOD, GST, and NQO1 (11). HO-1 catalyzes the degradation of heme into CO, bilirubin, and iron. Bilirubin shows strong antioxidant activity, whereas carbon monoxide inhibits inflammatory responses and apoptosis. GPX4 specifically eliminates lipid peroxides and suppresses ferroptosis. SOD converts superoxide into H_2_O_2_, which is further decomposed by catalase, thereby maintaining redox balance (12).

Excessive oxidative stress is a central factor driving OP, and dysfunction of Nrf2, the main antioxidant defense system of the body, is closely associated with OP (13). In the OP microenvironment, excessive ROS disrupts bone metabolism through several mechanisms: ROS induces mitochondrial dysfunction in osteoblasts and activates caspase-3/9-mediated intrinsic apoptosis. ROS inhibits RUNX2, thereby reducing OCN and COL1A1 synthesis (14). ROS promotes the differentiation of BMMs into mature osteoclasts. ROS upregulates TRAP, CTSK, and MMP-9, thereby enhancing osteoclastic resorption (15). ROS impairs the self-renewal and osteogenic capacity of BMSCs, further aggravating bone imbalance (16).

**Figure 1:**
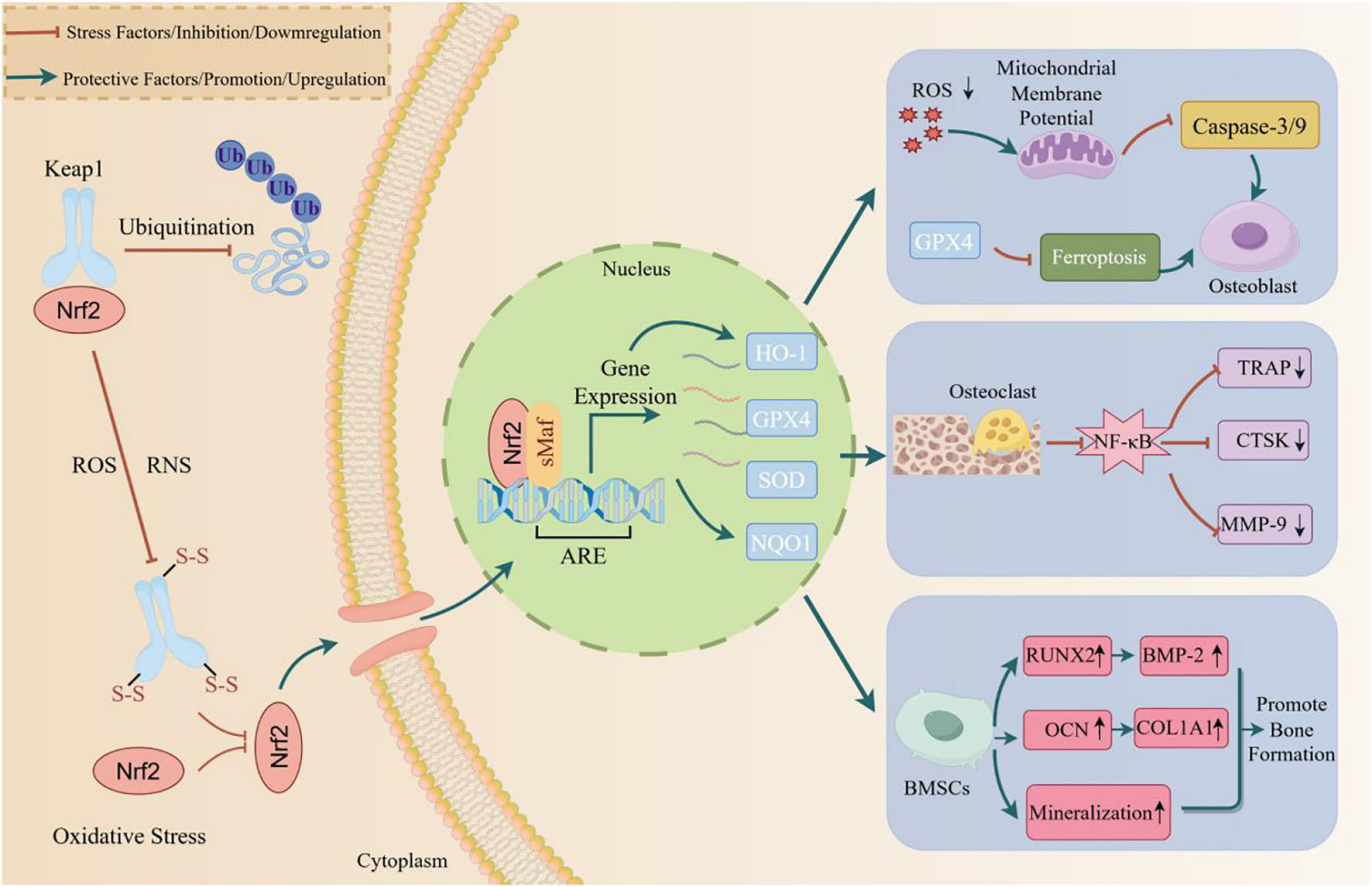
Molecular mechanisms of the Nrf2 pathway and its role in OP. This illustrates Nrf2 activation: under oxidative stress, Nrf2 dissociates from Keap1, translocates to the nucleus, binds AREs, and induces HO-1, SOD, and GPX4, scavenging ROS, reducing oxidative damage, and inhibiting osteoblast apoptosis, thereby protecting bone, promoting formation, and delaying bone loss. (Created by the authors using Figdraw).

The Nrf2 pathway regulates bone metabolism through several mechanisms and produces anti-OP effects: First, activation of Nrf2 increases antioxidant proteins such as HO-1, GPX4, and SOD, removing excessive ROS in osteoblasts and BMSCs. This suppresses mitochondrial apoptotic pathways and ferroptosis, thereby preserving the survival and function of cells related to bone formation (17). Studies have confirmed that Nrf2 knockout (Nrf2-/-) mice show spontaneous OP phenotypes, including reduced bone mass, damaged trabecular microarchitecture, decreased osteoblast numbers, and increased osteoclast activity, together with markedly elevated sensitivity to oxidative stress injury. Intervention with Nrf2 activators (e.g., sulforaphane) significantly improves bone loss in OVX mice, increases antioxidant protein expression in bone tissue, inhibits osteoblast apoptosis, and reduces osteoclast activity (7). Second, Nrf2 decreases osteoclast differentiation and bone resorption by suppressing ROS generation and NF-κB pathway activation in osteoclasts (18). *In vitro*, Nrf2 activation downregulates TRAP/CTSK/MMP-9 in BMMs, decreasing osteoclast numbers and resorption pits through ROS scavenging and NF-κBp65 inhibition (19). Third, Nrf2 promotes the differentiation of bone marrow mesenchymal stem cells (BMSCs) into osteoblasts and enhances bone formation. Nrf2 directly regulates RUNX2 and activates BMP-2/Smad signaling, thereby promoting BMSC osteogenesis and mineralization (20) (**Figure 1**).

### 2.2 Molecular Mechanisms of the NF-κB Pathway and Its Role in OP

**Figure 2:**
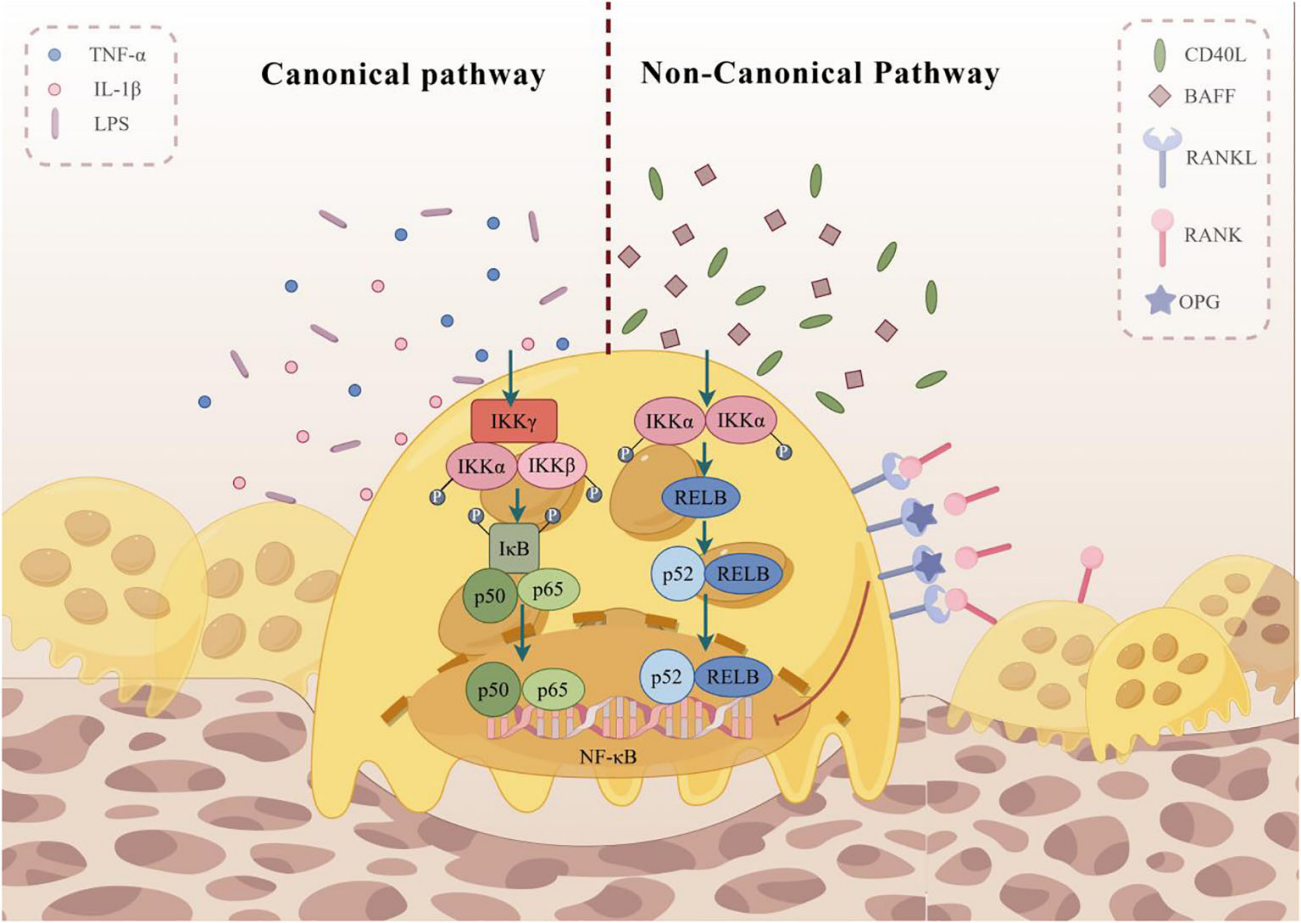
Molecular mechanisms of the NF-κB pathway and its role in OP.!!!Both canonical and non-canonical NF-κB signaling cascades are illustrated, detailing the activation of IKK complexes, IκB degradation, and nuclear translocation of p65/p50 and p52/RelB dimers. Canonical NF-κB activation predominantly drives pro-inflammatory cytokine production and osteoclast differentiation, while non-canonical signaling contributes to immune regulation and bone remodeling. Together, these pathways promote chronic inflammation, excessive osteoclastogenesis, and enhanced bone resorption, thereby accelerating the progression of OP.(Created by the authors using Figdraw).

NF-κB is a family of nuclear transcription factors consisting of five subunits: p65 (RelA), p50, p52, c-Rel, and RelB. Each subunit contains an RHD and forms homo/heterodimers (p65/p50 is the most common) for gene regulation (21). Under physiological conditions, NF-κB dimers bind to IκB proteins and remain inactive in the cytoplasm (22). IκB contains an ankyrin repeat that binds the NF-κB RHD and masks nuclear localization signals (23). NF-κB activation occurs through canonical and non-canonical pathways: Canonical pathway: activated by TNF-α, IL-1β, and LPS. It activates the IKK complex, which phosphorylates IκBα at Ser32/36. IκBα is subsequently ubiquitinated and degraded. NF-κB dimers (mainly p65/p50) translocate into the nucleus and bind κB sites to regulate inflammation, immunity, and osteoclast-related genes (24). The non-canonical pathway is mainly activated by stimuli such as CD40L and BAFF. It induces IKKα homodimer activation, causing phosphorylation and processing of the p100 precursor protein into p52. p52/RelB translocates into the nucleus and regulates lymphocyte development and immunity, with relatively minor effects on bone (25). NF-κB is a central link between chronic inflammation and bone imbalance; its excessive activation is important in OP pathogenesis (26). In OP, NF-κB regulates bone metabolism through multiple mechanisms. First, it regulates the RANKL/RANK/OPG axis to promote osteoclast activation and bone resorption. RANKL (receptor activator of nuclear factor kappa-B ligand), RANK (receptor activator of nuclear factor kappa-B), and OPG (osteoprotegerin) constitute a core axis controlling osteoclast differentiation. RANKL binds to RANK on osteoclast surfaces and activates downstream signaling pathways to promote osteoclast differentiation. OPG acts as a soluble receptor and binds to RANKL, blocking RANK-RANKL interaction and suppressing osteoclast activation (27). The NF-κB pathway can increase RANKL expression in bone marrow stromal cells, osteoblasts, and immune cells while decreasing OPG expression, thereby elevating the RANKL/OPG ratio. This activates the NF-κB pathway in osteoclasts and forms a positive feedback loop that promotes osteoclast precursor differentiation, maturation, and survival, ultimately increasing bone resorption activity (28). *In vitro*, inhibition of IKKβ downregulates RANKL, upregulates OPG, and reduces osteoclasts and resorption pits (29). Second, it suppresses osteoblast proliferation and differentiation, thereby reducing bone formation. Excessively activated NF-κB inhibits osteoblast function through direct and indirect pathways: NF-κB binds RUNX2 and suppresses OCN/COL1A1/ALP transcription and osteoblast differentiation (30). NF-κB promotes the secretion of TNF-α, IL-1β, and IL-6. These mediators activate the MAPK pathway, induce osteoclast apoptosis, and further inhibit bone formation (31). Studies have shown that NF-κBp65 knockout mice exhibit markedly enhanced osteoblast differentiation capacity and significantly increased bone mass. Conversely, in OVX mouse models, p65 protein expression is significantly upregulated, accompanied by reduced osteoblast numbers and weakened bone matrix synthesis capacity. Inhibition of p65 expression significantly improves bone metabolic imbalance (32). Third, it induces a chronic inflammatory microenvironment, thereby worsening bone metabolic disorders. The NF-κB pathway can establish a persistent chronic inflammatory microenvironment through a positive feedback loop of inflammatory factors, further promoting OP progression (33). Activated NF-κB enhances the transcriptional expression of inflammatory factors such as TNF-α, IL-1β, IL-6, and IL-8. which in turn reactivate the NF-κB pathway while also activating the MAPK pathway. This generates a vicious cycle involving the “cytokine-NF-κB/MAPK pathway” axis, which not only intensifies osteoclast activation and osteoblast injury but also impairs the osteogenic differentiation potential of BMSCs, thereby further disturbing bone metabolic equilibrium (34). Chronic inflammation induces oxidative stress, which further activates NF-κB and MAPK through ROS. This forms multiple overlapping pathological mechanisms that accelerate OP progression (35) (**Figure 2**).

### 2.3 Molecular Mechanisms of the MAPK Pathway and Its Role in OP

**Figure 3:**
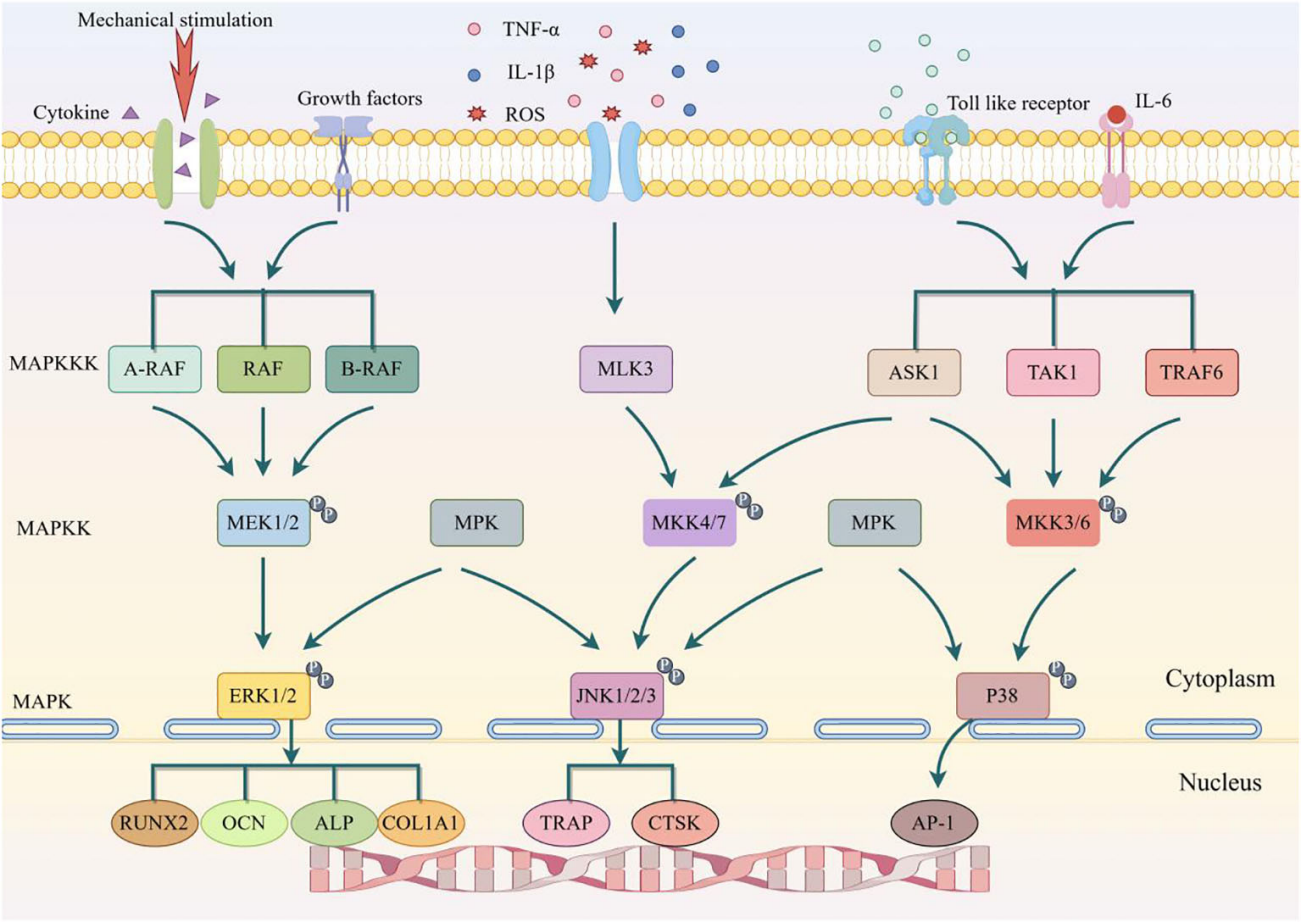
Molecular mechanisms of the MAPK pathway and its role in OP. The figure presents the signaling pathways and functional differences among the three major classical MAPK branches: ERK, JNK, and p38. Specifically, the ERK pathway primarily promotes osteoblast proliferation and differentiation and positively regulates bone formation; the JNK and p38 pathways are often activated by oxidative stress and inflammatory signals, primarily mediating osteoblast apoptosis, exacerbating inflammatory damage, and promoting bone resorption; simultaneously, the p38 MAPK exhibits a bidirectional regulatory characteristic dependent on activation intensity, whereby different levels of activation can produce diametrically opposed effects on bone metabolism regulation.(Created by the authors using Figdraw).

MAPK is an important signal transduction pathway that connects extracellular stimuli with nuclear responses. Through a three-level kinase cascade, it converts extracellular signals into transcriptional responses and regulates proliferation, differentiation, apoptosis, and inflammation (36). MAPK includes three classical branches: ERK1/2, JNK, and p38 MAPK, as well as minor branches (ERK5, NLK) that have distinct functions in bone (37) (**Figure 3**).

ERK1/2 is activated by growth factors, mechanical stress, and cytokines. Activation: Raf→MEK1/2→ERK1/2 (Thr202/Tyr204). Activated ERK1/2 translocates into the nucleus and phosphorylates Elk-1, c-Fos, and c-Myc (38). The ERK1/2 pathway mainly promotes osteogenesis in bone metabolism: on the one hand, ERK1/2 activation increases osteogenic marker genes such as RUNX2, OCN, ALP, and COL1A1, supporting BMSC differentiation into osteoblasts, osteoblast proliferation, and mineralization of the bone matrix (39); BMP-2 activates ERK1/2, thereby increasing ALP activity, nodule formation, and RUNX2/OCN expression. These effects are reversed by the MEK1/2 inhibitor U0126 (40). Conversely, the ERK1/2 pathway suppresses osteoclast apoptosis and maintains osteoclast activity, although this regulatory effect is weaker than that of the JNK and p38 MAPK pathways. Under physiological conditions, it preserves the dynamic balance between bone resorption and bone formation (41). In OP models, ERK1/2 expression is reduced, leading to decreased osteoblast differentiation and bone formation. Activation of the ERK1/2 pathway markedly improves bone loss in ovariectomized (OVX) mice (42).

JNK is activated by ROS, inflammatory factors such as TNF-α and IL-1β, and ultraviolet radiation. Activation: ASK1/MLK3→MKK4/7→JNK (Thr183/Tyr185). Activated JNK enters the nucleus and phosphorylates c-Jun, ATF2, and Elk-1 (43). JNK mainly contributes to bone injury in OP. First, it induces osteoblast apoptosis and suppresses osteoblast differentiation. Stimuli such as ROS and TNF-α activate the JNK pathway, phosphorylate Bcl-2 family proteins (e.g., Bad, Bax), promote mitochondrial cytochrome c release, activate the caspase apoptotic pathway, and eventually cause osteoblast apoptosis. Meanwhile, JNK inhibits the transcriptional activity of RUNX2, downregulates osteogenic marker gene expression, and suppresses osteoblast differentiation (44). Studies have shown that JNK1 knockout mice are significantly resistant to OVX-induced bone loss, with increased osteoblast numbers and reduced apoptosis rates (45). Second, the JNK pathway enhances osteoclast activation and bone resorption. RANKL activates the JNK pathway to promote the differentiation of osteoclast precursors into mature osteoclasts, upregulate osteoclast marker genes such as TRAP and CTSK, and strengthen bone resorption activity. In addition, the JNK pathway amplifies osteoclast activation signals by regulating the NF-κB pathway (46).

p38 MAPK is activated by inflammation, oxidative stress, and mechanical stress. Activation: ASK1/TAK1→MKK3/6→p38 (Thr180/Tyr182). Activated p38 moves into the nucleus and phosphorylates ATF2, CHOP, and Elk-1 (47). p38 has dual functions in bone, depending on activation intensity, stimulus type, and cell type: under low-intensity and short-term activation, p38 MAPK promotes osteoblast differentiation and bone formation by increasing RUNX2 and OCN expression, thereby enhancing osteogenic differentiation of BMSCs; however, high-intensity and prolonged activation aggravates bone injury by inducing inflammatory cytokine secretion and promoting osteocyte apoptosis (48). In osteoclast regulation, p38 MAPK promotes osteoclast precursor differentiation and increases bone resorption activity by activating the NF-κB pathway and AP-1 (activator protein-1) (49). In OP, p38 is excessively activated, promoting inflammation, osteoblast apoptosis, and osteoclast activation. Moderate inhibition of p38 improves bone microarchitecture and mechanical properties (50).

### 2.4 Interactions Among Nrf2, NF-κB, and MAPK

**Figure 4:**
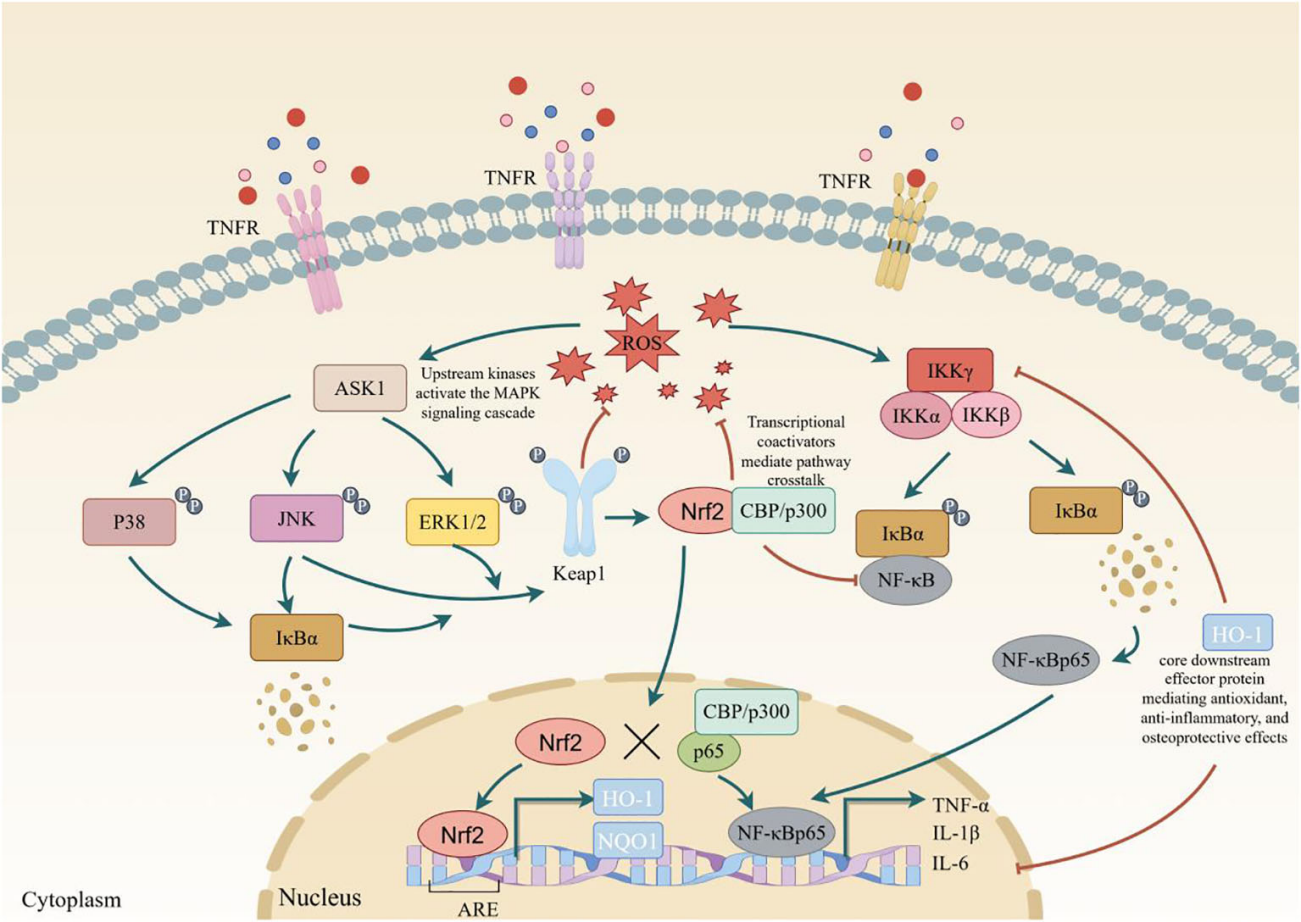
Interactions among Nrf2, NF-κB, and MAPK. This figure integrates the cross-talk relationships among these three core signaling pathways and clearly illustrates the functional roles of key hub molecules such as ASK1, CBP/p300, and HO-1. Among these, ASK1 functions as a key upstream kinase to initiate the MAPK cascade; CBP/p300 mediates transcriptional crosstalk between Nrf2 and NF-κB within the nucleus; and HO-1, as a common downstream effector protein, simultaneously mediates antioxidant and anti-inflammatory effects; Through upstream signal coordination, nuclear transcriptional interactions, and downstream effector synergy, these molecules collectively form a complex regulatory network that jointly participates in the regulation of bone metabolic homeostasis and the pathogenesis of OP. (Created by the authors using Figdraw).

Nrf2, NF-κB, and MAPK do not function separately. They establish crosstalk networks through oxidative stress, inflammation, and transcription factors, jointly regulating bone homeostasis. The main intersections are as follows (**Figure 4**).

#### 2.4.1 Oxidative stress as the core hub of cross-talk among the three pathways

Excessive ROS serve as upstream activators of MAPK and NF-κB: ROS oxidize MAPKKK cysteines and activate JNK and p38; ROS also activate IKK and induce NF-κB (51). Activated MAPK further regulates Nrf2 and NF-κB: p38/JNK phosphorylate Keap1 and enhance Nrf2 nuclear translocation; p38/JNK phosphorylate IκBα and promote NF-κB activation (52). Nrf2 removes ROS, thereby inhibiting MAPK/NF-κB and forming a negative feedback loop (53). Nrf2-/- mice show elevated MAPK/NF-κB activity, increased ROS/inflammation and more severe bone damage. Nrf2 activation reduces p38/JNK/ERK/NF-κB phosphorylation and ROS/inflammation in OVX mice (54).

#### 2.4.2 Inflammatory cytokines serve as key mediators in the cross-talk among three pathways

NF-κB activation induces the secretion of TNF-α, IL-1β and IL-6. These cytokines reactivate MAPK/NF-κB signaling, thereby amplifying inflammatory responses. They also activate MAPK, promoting Nrf2 nuclear translocation and antioxidant defense (55). Furthermore, the Nrf2 pathway can negatively regulate inflammatory responses by suppressing NF-κB pathway activity and reducing inflammatory cytokine secretion. Nrf2 competes with NF-κBp65 for transcriptional coactivators (e.g., CBP/p300), thereby inhibiting NF-κB binding to target genes. The Nrf2 target HO-1 inhibits IKK through CO, thereby blocking NF-κB activation (56).

#### 2.4.3 Transcriptional factor interactions form the molecular basis where three pathways converge

MAPK-phosphorylated ERK/p38 directly phosphorylate Nrf2 at Ser40, enhancing its nuclear translocation. MAPK also phosphorylates NF-κBp65 at Ser536, thereby increasing its activity (57). Nrf2 and NF-κB show mutual antagonism. In addition to competing for coactivators, Nrf2 inhibits NF-κB pathway activation by upregulating the gene expression of HO-1, NQO1, and other molecules. Conversely, NF-κB suppresses Nrf2 pathway activity by regulating Nrf2 transcription and ubiquitin-mediated degradation (58). This antagonistic relationship preserves the dynamic balance among the three pathways and coordinates bone metabolism, oxidative stress, and inflammatory responses. Such crosstalk is critical in the pathogenesis of OP. TCM targets key nodes within this network, restores homeostasis, and regulates bone metabolism. This approach is highly consistent with the characteristic “multi-target, multi-pathway” mechanism of action of TCM (6). As shown in **Figure 4**, ASK1, CBP/p300, and HO-1 mediate pathway crosstalk at the levels of upstream signaling, nuclear transcription and downstream function. This clearly defines the specific modes of action of molecules at each crosstalk node, providing an intuitive basis for understanding the intrinsic mechanisms through which multiple pathways synergistically regulate bone metabolism.

## 3 Single-component traditional Chinese medicines targeting the Nrf2/NF-κB/MAPK pathway to intervene in OP

**Figure 5:**
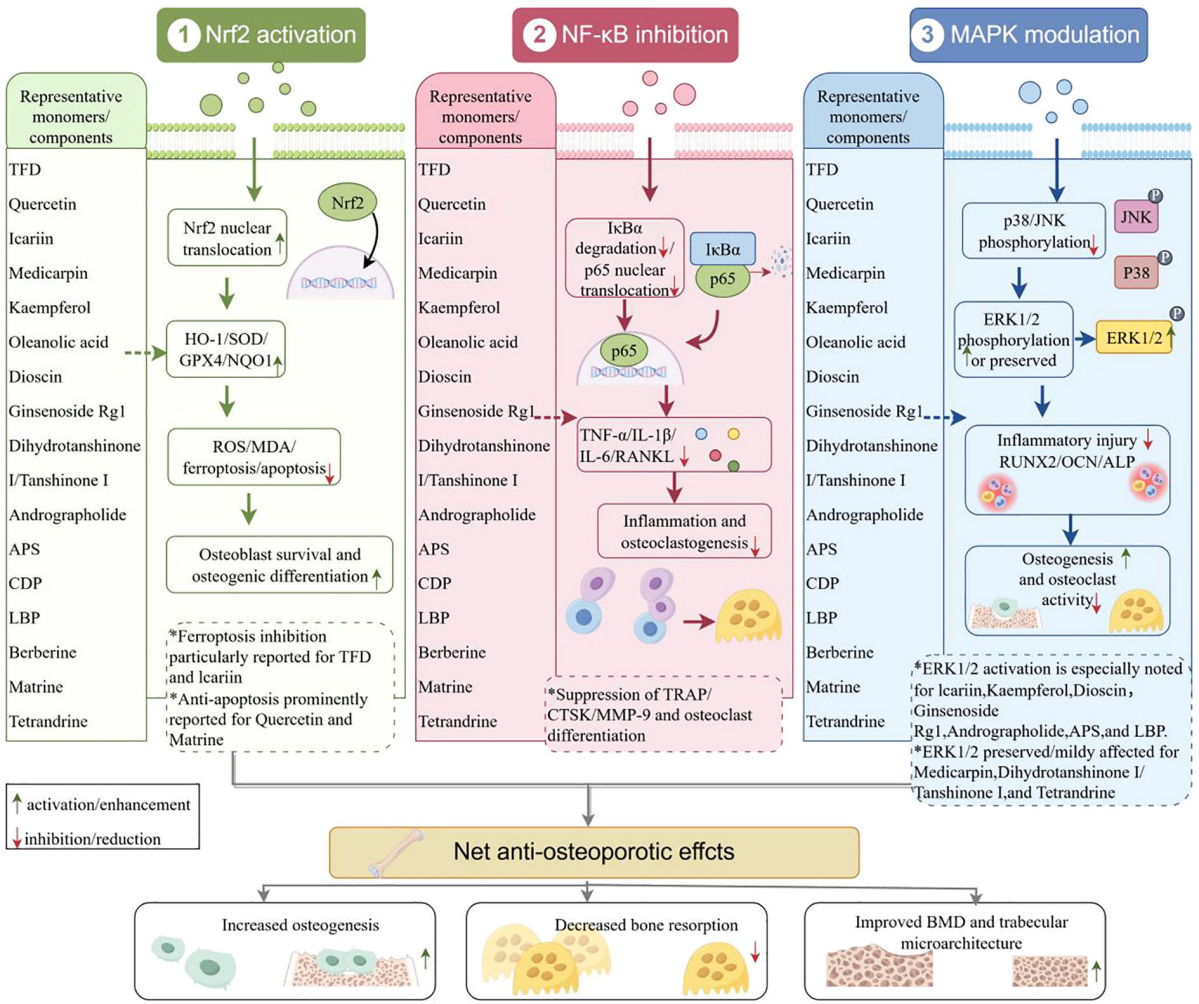
Schematic diagram of representative TCM monomers targeting the Nrf2/NF-κB/MAPK signaling axis in osteoporosis treatment. (Created by the authors using Figdraw).

Based on the pathway background described above, this section focuses on clarifying the mechanisms by which different TCM compounds target the Nrf2/NF-κB/MAPK pathway to exert anti-osteoporotic effects. Numerous TCM monomers (e.g., flavonoids, terpenoids, polysaccharides, alkaloids) have been shown to produce anti-osteoporosis effects by regulating the Nrf2/NF-κB/MAPK pathway. Their relatively clear mechanisms of action and defined target sites provide important support for investigating the anti-osteoporosis mechanisms of TCM and for developing new drugs. TCM monomers synergistically regulate bone metabolic homeostasis by acting on the Nrf2, NF-κB, and MAPK signaling pathways. Their main modes of action and the positions of representative monomers are shown in **Figure 5**.

### 3.1 Flavonoids

Flavonoids are a rich and biologically active class of secondary metabolites in TCM, with various pharmacological activities, including antioxidant, anti-inflammatory, anti-apoptotic, and bone metabolism-regulating effects. Their anti-OP effects are mainly related to regulation of the Nrf2/NF-κB/MAPK pathway.

#### 3.1.1 Total flavonoids of *Drynaria fortunei*

TFD is the major active component of Drynaria fortunei, mainly consisting of monomeric compounds such as naringin, neohesperidin, and drynarin. It has the effects of tonifying the kidneys, strengthening bones, promoting blood circulation, and relieving pain (59). TFD has now entered the clinical research stage for OP. Several randomized controlled trials (RCTs) and systematic reviews have confirmed that it significantly improves bone mineral density in the lumbar spine and femoral neck of patients with primary OP, relieves bone pain symptoms, and shows a favorable safety profile. Related preparations have already been applied in clinical practice as adjunctive therapies (60). Studies suggest that TFD activates the Nrf2 pathway to suppress oxidative stress and ferroptosis in osteoblasts, thereby promoting bone formation. In a rat model of diabetes-induced OP established using a high-sugar, high-fat diet combined with streptozotocin, 12 weeks of oral TFD treatment significantly increased femoral bone mineral density (BMD), bone volume fraction (BV/TV), and trabecular thickness (Tb.Th), while markedly decreasing trabecular separation (Tb.Sp). The mRNA and protein expression levels of Nrf2, HO-1, and GPX4 in bone tissue were significantly upregulated, whereas intracellular iron ion, ROS, and MDA levels were significantly decreased. The expression of ferroptosis markers (ACSL4, PTGS2) in BMSCs was also markedly downregulated (20). *In vitro* experiments showed that TFD significantly improved the survival rate of high-glucose-induced BMSCs, enhanced ALP activity, promoted mineralized nodule formation, and upregulated RUNX2 and OCN expression. In addition, studies showed that Nrf2 silencing by lentiviral transfection markedly weakened the anti-ferroptotic and osteogenesis-promoting effects of TFD, confirming that its action depends on the Nrf2 pathway (61). Furthermore, TFD reduces inflammatory responses and osteoclast activation by inhibiting the NF-κB and MAPK pathways: TFD significantly downregulates TNF-α, IL-1β, and IL-6 expression in the bone tissue of OVX mice, while suppressing NF-κB p65 phosphorylation and nuclear translocation. Simultaneously, TFD inhibited p38 and JNK phosphorylation while promoting ERK1/2 phosphorylation, thereby restoring osteoblast function and suppressing osteoclast differentiation (62).

#### 3.1.2 Quercetin

Quercetin is a flavonoid component widely distributed in traditional Chinese medicines such as Ligustrum lucidum, Crataegus pinnatifida, and Ginkgo biloba leaves, and it exhibits multiple activities, including antioxidant, anti-inflammatory, and anti-osteoporotic effects (63). To date, quercetin has only been assessed in small-scale observational studies and preliminary exploratory trials; no standardized clinical trials specifically targeting OP have been performed, and the clinical evidence remains relatively limited. Research indicates that quercetin alleviates oxidative stress injury and promotes osteoblast differentiation by activating the Nrf2/HO-1 pathway. In a hydrogen peroxide-induced osteoblast (MC3T3-E1) injury model, quercetin treatment significantly increased cell survival rates, markedly decreased ROS and MDA levels, and substantially enhanced SOD and GSH activity. The mRNA and protein expression levels of Nrf2 and HO-1 were significantly upregulated, and the nuclear translocation capacity of Nrf2 was clearly enhanced. Meanwhile, quercetin significantly inhibited hydrogen peroxide-induced osteoblast apoptosis by upregulating Bcl-2 expression, downregulating Bax and caspase-3 expression, and promoting bone mineralization nodule formation through the upregulation of RUNX2, OCN, and ALP expression (64, 65). In osteoclast regulation, quercetin inhibits osteoclast differentiation by suppressing the NF-κB and MAPK pathways: it significantly reduces RANKL-induced differentiation of BMMs into osteoclasts, downregulates TRAP, CTSK, and MMP-9 expression, and decreases the number of resorption pits. Its mechanism involves inhibiting IκBα degradation and NF-κB p65 nuclear translocation, as well as suppressing p38 and JNK phosphorylation (66). *In vivo* studies showed that 12 weeks of oral quercetin administration in OVX mice significantly increased femoral BMD, BV/TV, and Tb.Th, while markedly reducing the number of osteoclasts. Bone tissue showed upregulated Nrf2 and HO-1 expression, together with downregulated NF-κBp65, p38, and JNK phosphorylation levels (67).

#### 3.1.3 Icariin

Icariin, the main active flavonoid component of Epimedium, has kidney-tonifying and yang-strengthening properties, as well as bone-strengthening effects. It is a commonly used Chinese herbal medicine component for the clinical prevention and treatment of OP (68). Icariin has been examined in several randomized controlled trials for treating postmenopausal osteoporosis (PMOP). Studies have shown that oral administration of 60 mg icariin per day for 24 months can effectively slow bone loss. In addition, treatment with 740 mg total flavonoids of Epimedium (rich in icariin) daily for six weeks markedly increases the level of the bone formation marker BSAP and improves bone metabolic status, with good clinical safety (69). Research suggests that icariin exerts anti-osteoporosis effects by regulating the Nrf2/NF-κB/MAPK pathways. In an ovariectomized rat model, icariin intervention significantly increased lumbar spine and femoral BMD and clearly improved bone microarchitecture. The expression of Nrf2, HO-1, and SOD in bone tissue was significantly upregulated, whereas ROS and MDA levels were significantly reduced. Meanwhile, icariin inhibited NF-κB p65 phosphorylation and nuclear translocation, downregulated TNF-α and IL-1β expression, and suppressed osteoclast activation (70–72). *In vitro* experiments showed that icariin promotes MC3T3-E1 cell proliferation and differentiation, enhances ALP activity, facilitates mineralized nodule formation, and upregulates RUNX2 and OCN expression. Its mechanism involves activating the ERK1/2 pathway, promoting Nrf2 nuclear translocation, and inhibiting the p38, JNK, and NF-κB pathways (73). Furthermore, icariin can inhibit ferroptosis in BMSCs by activating the Nrf2 pathway, thereby promoting osteogenic differentiation and providing an adequate cellular source for bone formation (74).

#### 3.1.4 Medicarpin

Medicarpin is a representative active pterocarpan constituent derived from Cajanus cajan of the Fabaceae family. It exerts multiple osteoprotective pharmacological activities, including anti-inflammatory and antioxidant effects, suppression of abnormal osteoclast differentiation, and maintenance of physiological osteoblast functions, and has attracted considerable attention in natural drug research against OP in recent years (75). Accumulating pharmacological evidence has confirmed that medicarpin produces osteoprotective effects through multi-target regulation of the interactive Nrf2/NF-κB/MAPK signaling network. Regarding antioxidant activity and osteogenesis promotion, it effectively activates the Nrf2 signaling pathway, upregulates the expression of endogenous antioxidant proteins such as HO-1 and SOD, removes excessive intracellular reactive oxygen species, alleviates oxidative stress-induced bone tissue injury, and further inhibits osteoblast apoptosis (75). In terms of anti-inflammatory activity and osteoclastogenesis inhibition, medicarpin markedly blocks activation of the NF-κB signaling pathway, restrains IκBα phosphorylation and degradation, inhibits p65 nuclear translocation and transcriptional activity, and downregulates key markers related to osteoclast differentiation, including RANKL, TRAP, CTSK and MMP-9. Therefore, it notably suppresses RANKL-induced differentiation of bone marrow monocytes into osteoclast precursors and weakens the bone resorption capacity of osteoclasts (76). Furthermore, this compound selectively inhibits excessive phosphorylation of the inflammation-related p38 and JNK sub-pathways, reduces the secretion of pro-inflammatory cytokines and improves the local inflammatory microenvironment in bone tissues, while showing no obvious inhibitory effect on the ERK1/2-mediated osteogenic signaling pathway, thereby maintaining the dynamic balance between osteogenesis promotion and inflammation inhibition regulated by MAPK family pathways (77). Further *in vitro* cellular experiments have verified that medicarpin inhibits the differentiation of bone marrow-derived macrophages into mature osteoclasts in a dose-dependent manner, reduces the formation of bone resorption pits, and downregulates the expression of osteoclast-specific functional proteins. Meanwhile, it alleviates osteoblast injury induced by oxidative stress and inflammatory stimuli and stabilizes the proliferation and differentiation functions of osteoblasts (76). In conclusion, medicarpin has definite action targets and comprehensive osteoprotective effects, making it a promising natural active monomer with considerable potential for new drug research and development as well as clinical application in the prevention and treatment of OP.

#### 3.1.5 Kaempferol

Kaempferol is a bioactive flavonoid widely distributed in many traditional Chinese medicines, including Kaempferia galanga, ginkgo leaves, Epimedium brevicornu, and sophora flower, and it exhibits notable antioxidant, anti-inflammatory, and bone metabolism-regulating activities (78). Accumulated studies have shown that kaempferol exerts multi-target regulatory effects on the Nrf2/NF-κB/MAPK signaling network. It activates the Nrf2 pathway to promote Nrf2 nuclear translocation and upregulate the expression of antioxidant proteins such as HO-1, SOD, and NQO1, thereby eliminating reactive oxygen species, alleviating oxidative stress injury, protecting mitochondrial function, and suppressing osteoblast apoptosis (79). Meanwhile, it inhibits the NF-κB pathway by blocking IκBα phosphorylation and degradation and restraining p65 nuclear translocation, and downregulates osteoclast-related genes, including RANKL, TRAP, CTSK, and MMP-9, to hinder osteoclast differentiation and bone resorption (80). In addition, it selectively modulates the MAPK pathway, inhibits excessive activation of p38 and JNK to reduce the release of pro-inflammatory factors, and moderately activates ERK1/2 signaling to promote osteoblast proliferation and differentiation as well as upregulate RUNX2 and OCN expression (78). *In vitro* experiments have confirmed that kaempferol can promote osteogenic differentiation of BMSCs, enhance ALP activity, accelerate mineralized nodule formation, and inhibit RANKL-induced osteoclast formation (81). *In vivo* studies based on OVX animal models have shown that kaempferol can markedly increase bone mineral density, improve trabecular bone microarchitecture, and reduce bone turnover rate. Accordingly, kaempferol is considered a promising flavonoid monomer for the prevention and treatment of OP (82).

### 3.2 Terpenoid compounds

Triterpenoids represent an important group of bioactive compounds in TCM. According to molecular structure, they are classified as monoterpenes, sesquiterpenes, diterpenes, triterpenes, and steroidal triterpenes. Their anti-osteoporosis effects are mainly related to regulation of the NF-κB/MAPK pathway, inhibition of inflammatory responses, and promotion of osteoblast function (83).

#### 3.2.1 Oleanolic acid

OA is a triterpene compound found in TCMs such as Ligustrum lucidum, Forsythia suspensa, and Crataegus pinnatifida, and shows anti-inflammatory, antioxidant, and bone metabolism-regulating activities (84). *In vitro* studies have confirmed that OA exerts bidirectional regulation of bone metabolism by inhibiting activation of the NF-κB signaling pathway: it significantly suppresses osteoclast differentiation and activation, while effectively promoting osteoblast proliferation and functional maturation. In a TNF-α-induced MC3T3-E1 osteoblast injury model, OA intervention markedly reversed the inhibitory effect of TNF-α on cell proliferation, increased cell survival rates, and significantly upregulated the mRNA and protein expression of RUNX2, OCN, and COL1A1, while enhancing ALP activity to promote osteogenic differentiation. Further mechanistic studies showed that OA alleviates inflammatory injury in osteoblasts by blocking TNF-α-mediated NF-κBp65 nuclear translocation, thereby markedly downregulating IL-6 and TNF-α expression (85). In osteoclast culture systems, OA significantly inhibited RANKL-induced differentiation of bone marrow-derived monocytes (BMMs) into osteoclasts, reduced the number of TRAP-positive cells and the bone resorption pit area, and downregulated TRAP, CTSK, and MMP-9 expression. This mechanism was related to inhibition of IκBα phosphorylation and degradation, thereby blocking activation of the NF-κB pathway (86). *In vivo* experiments demonstrated that OA gavage intervention in OVX mice significantly increased femoral BMD, markedly improved trabecular microarchitecture, substantially reduced osteoclast numbers, significantly downregulated NF-κBp65 phosphorylation levels in bone tissue, and markedly upregulated Nrf2 and HO-1 expression (87). In addition, OA modulates the MAPK pathway by inhibiting p38 and JNK phosphorylation, reducing osteocyte apoptosis, and synergistically producing anti-osteoporotic effects (88, 89).

#### 3.2.2 Dioscin

Dioscin is a steroidal triterpene component found in traditional Chinese medicines such as Chinese yam, Smilax glabra, and Polygonatum. It has effects including tonifying the kidneys and replenishing essence, strengthening tendons and bones, and exerting anti-inflammatory and antioxidant activities (90). Research has confirmed that dioscin produces anti-osteoporotic effects by regulating the Nrf2/NF-κB/MAPK pathway. In a rat OP model, after diosgenin intervention, rat femoral BMD, BV/TV, and Tb.Th were significantly increased, Tb.Sp was significantly decreased, and biomechanical properties (maximum load, elastic modulus) were significantly improved; the mRNA and protein expression levels of Nrf2 and HO-1 in bone tissue were significantly upregulated, whereas ROS and MDA levels were significantly reduced, and SOD and GSH activities were significantly increased (91, 92). Concurrently, diosgenin significantly inhibited NF-κB p65 phosphorylation and nuclear translocation, downregulated TNF-α, IL-1β, and IL-6 expression, and reduced osteoclast numbers. In addition, diosgenin promoted ERK1/2 phosphorylation while inhibiting p38 and JNK phosphorylation, upregulated RUNX2 and OCN expression, and promoted osteoblast differentiation (93). *In vitro* experiments demonstrated that diosgenin promotes osteogenic differentiation of BMSCs, enhances ALP activity, and facilitates bone-like nodule formation. Silencing of the Nrf2 gene significantly weakened both the osteogenesis-promoting and antioxidant effects of diosgenin, confirming that its action depends on the Nrf2 pathway (72, 94).

#### 3.2.3 Ginsenoside Rg1

Ginsenoside Rg1 is the main active diterpenoid component in ginseng and exhibits strong effects, such as replenishing vital energy and enhancing immunity (95). Research indicates that ginsenoside Rg1 alleviates oxidative stress injury by activating the Nrf2 pathway, thereby regulating the differentiation fate of bone marrow BMSCs. It promotes their differentiation into osteoblasts while inhibiting adipocyte differentiation, ultimately improving age-related bone metabolic imbalance. In both *in vitro* cellular experiments and *in vivo* aging-related bone loss models, ginsenoside Rg1 intervention significantly upregulated Nrf2 and HO-1 expression in bone tissue, markedly reduced ROS levels, and elevated the expression of osteoblast differentiation-related markers. This effectively reversed oxidative stress-mediated inhibition of osteogenic differentiation and adipogenic tendency, thereby delaying the progression of bone loss (96). *In vitro* experiments showed that ginsenoside Rg1 promotes osteogenic differentiation in aged BMSCs by upregulating RUNX2 and OCN expression. This mechanism involves activation of the ERK1/2 pathway, promotion of Nrf2 nuclear translocation, and inhibition of p38 MAPK and NF-κB pathway activity (97). In addition, ginsenoside Rg1 reduces osteocyte apoptosis by inhibiting the JNK pathway while concurrently suppressing osteoclast differentiation, thereby synergistically restoring bone metabolic balance (98).

#### 3.2.4 Dihydrotanshinone I and tanshinone I (*Salvia miltiorrhiza*)

Salvia miltiorrhiza is a classic TCM used for activating blood circulation and removing blood stasis. Dihydrotanshinone I and tanshinone I are characteristic lipophilic diterpenoid constituents of this herb and possess strong anti-inflammatory, antioxidant, pro-angiogenic, and osteoprotective activities (99). Recent studies have verified that both compounds can precisely target the Nrf2/NF-κB/MAPK signaling pathways. They activate Nrf2 signaling to upregulate HO-1 and SOD expression, effectively eliminate reactive oxygen species, relieve oxidative stress, protect mitochondrial function, and inhibit osteoblast apoptosis (100). Meanwhile, they suppress the NF-κB pathway by blocking IKKα/β activation and IκB degradation, inhibiting p65 nuclear translocation, and downregulating key osteoclastogenic factors, including RANKL, TRAP, CTSK, and MMP-9, thereby markedly restraining RANKL-induced differentiation of osteoclast precursors and reducing bone resorption activity (101). In addition, they selectively inhibit excessive phosphorylation of p38 and JNK to reduce pro-inflammatory cytokine secretion and osteoblast apoptosis, while exerting negligible effects on the ERK1/2-mediated osteogenic pathway, thereby maintaining the balance between osteogenesis promotion and inflammation inhibition mediated by MAPK signaling (100). *In vitro* experiments indicate that dihydrotanshinone I and tanshinone I significantly inhibit RANKL-induced differentiation of bone marrow-derived macrophages into osteoclasts, reduce the formation of bone resorption pits, and downregulate osteoclast-specific markers. They also alleviate osteoblast damage caused by oxidative stress, suppress cell apoptosis, and maintain osteogenic activity (102). *In vivo* studies further confirmed that these two ingredients can improve trabecular bone microarchitecture, reduce bone resorption, and alleviate inflammatory infiltration, serving as promising novel natural monomers against OP (100). In summary, because of their clear osteoprotective effects validated in cellular and animal experiments, dihydrotanshinone I and tanshinone I show considerable research and development potential for OP treatment. Nevertheless, four major clinical research gaps remain, including clinical efficacy evaluation, safe medication criteria, optimal dosage and dosage form, and screening of applicable populations. Current relevant research is still limited to basic experiments and lacks a complete clinical research system. Clinical evidence-based data guiding diagnosis and treatment remain insufficient, leaving considerable research prospects and evidence gaps for their clinical translation from basic pharmacological findings to practical prevention and treatment of OP.

#### 3.2.5 Andrographolide

Andrographolide is a representative diterpenoid active ingredient derived from Andrographis paniculata of the Acanthaceae family. It has notable anti-inflammatory, antioxidant, immunomodulatory, and osteoprotective effects, and is a well-studied natural terpenoid monomer targeting inflammation- and oxidative stress-related signaling pathways (103). Andrographolide precisely regulates the interactive network of the Nrf2/NF-κB/MAPK pathways. It strongly activates the Nrf2 pathway by promoting dissociation of the Keap1-Nrf2 complex and facilitating Nrf2 nuclear translocation, thereby markedly upregulating HO-1, SOD, and GPX4 expression, eliminating intracellular reactive oxygen species, reducing oxidative injury, and protecting osteoblasts and bone marrow mesenchymal stem cells from stress-induced damage (104). Meanwhile, it clearly inactivates the NF-κB pathway by blocking activation of the IKK complex, delaying IκBα degradation and suppressing p65 nuclear translocation, which further downregulates pro-inflammatory and osteoclastogenic factors, including TNF-α, IL-1β, and RANKL, and effectively restrains osteoclast differentiation and excessive bone resorption (104). Furthermore, it differentially regulates the MAPK pathway, inhibits abnormal phosphorylation of p38 and JNK to alleviate inflammatory injury, and moderately activates ERK1/2 signaling to promote osteogenesis, ultimately maintaining the dynamic homeostasis of bone metabolism (103). *In vitro* experiments have shown that andrographolide suppresses RANKL-induced osteoclast formation and reduces bone resorption pit formation in a dose-dependent manner, while improving the physiological function of osteoblasts and upregulating osteogenic marker expression (104, 105). *In vivo* studies based on ovariectomy-induced estrogen-deficient bone loss models have verified that andrographolide can effectively increase bone mineral density, restore trabecular bone microarchitecture and reduce inflammatory infiltration in bone tissues, exerting favorable osteoprotective effects through antioxidant activity, anti-inflammatory action and bidirectional regulation of osteogenesis and osteoclastogenesis (106). At present, relevant clinical investigations remain relatively limited, and most studies are still at the basic experimental stage. A small number of clinical observations indicate that it may help ameliorate bone metabolic disorders and local inflammatory conditions, whereas standardized combined medication regimens have not yet been established.

### 3.3 Polysaccharide components

Polysaccharides in TCM are an important class of water-soluble bioactive compounds. They show various activities, including immunomodulatory, antioxidant, anti-inflammatory, and bone metabolism-regulating effects. Their anti-osteoporotic effects are mainly associated with activation of the Nrf2 pathway and inhibition of the NF-κB/MAPK pathway, while showing low toxicity and high safety.

#### 3.3.1 Astragalus polysaccharide

APS is the major active polysaccharide component of Astragalus membranaceus, known for its effects of boosting qi, strengthening the spleen, tonifying the kidneys, and strengthening bones. It is widely used in the prevention and treatment of OP (107). Research indicates that APS alleviates oxidative stress injury and promotes bone formation by activating the Nrf2 pathway. In an OVX mouse OP model, oral APS intervention significantly increased femoral BMD, BV/TV, and Tb.Th, markedly improved trabecular microarchitecture, significantly upregulated the mRNA and protein expression of Nrf2, HO-1, and SOD in bone tissue, while reducing ROS and MDA levels and markedly decreasing osteoblast apoptosis rates (108). *In vitro* experiments demonstrated that APS promotes osteogenic differentiation of BMSCs, enhances ALP activity, facilitates mineralized nodule formation, and upregulates osteogenesis-related genes, such as RUNX2 and COL1A1. Concurrently, APS inhibits activation of the NF-κB signaling pathway in bone marrow mesenchymal stem cells, downregulates RANKL expression, and reduces the levels of osteoclast marker genes, including NFATc1 and TRAP, ultimately suppressing osteoclast differentiation in a dose-dependent manner (109). Further studies confirmed that APS promotes osteoblast function by activating the PI3K/Akt pathway to enhance Nrf2 nuclear translocation, while simultaneously regulating the MAPK pathway by promoting ERK1/2 phosphorylation and inhibiting p38 and JNK phosphorylation. This mechanism acts synergistically with Wnt/β-catenin pathway activation to exert combined anti-osteoporosis effects (109, 110).

#### 3.3.2 *Cistanche deserticola* polysaccharide

CDP, an active polysaccharide component of Cistanche deserticola, regulates metabolism, improves reproductive endocrine function, promotes bone metabolism, and enhances skeletal mechanical properties, making it suitable for preventing and treating age-related OP (111). Experimental studies indicate that CDP suppresses osteoclast differentiation by blocking the MAPK/NF-κB pathway while also promoting osteoblast differentiation. In the SAMP6 mouse model of aging, CDP intervention increased trabecular bone thickness, significantly elevated bone mineral density, markedly enhanced SOD enzyme activity, substantially reduced MDA levels, and clearly decreased oxidative stress levels (112). *In vitro* experiments revealed that CDP significantly upregulates the mRNA and protein expression of BMP-2, OPG, and RUNX2 in BMSCs, thereby promoting osteogenic differentiation. Simultaneously, CDP significantly inhibited RANKL-induced osteoclast differentiation in BMMs, downregulated TRAP, CTSK, NF-κBp65, p38, and JNK expression, and this mechanism was associated with blocking MAPK/NF-κB pathway activation (111).

#### 3.3.3 *Lycium barbarum* polysaccharide

LBP is the main active polysaccharide component in goji berries and exhibits regulatory effects on hepatic and renal metabolic functions, improves reproductive endocrine homeostasis, and protects retinal function. Its anti-osteoporosis activity is related to targeting the Nrf2/NF-κB/MAPK pathways (113, 114). In an OVX rat model, LBP intervention significantly increased BMD in the lumbar spine and femur, while markedly improving bone microstructural integrity and mechanical properties. Mechanistic studies indicate that LBP upregulates the expression of antioxidant-related proteins Nrf2 and HO-1 in bone tissue, thereby decreasing ROS and MDA levels and alleviating oxidative stress injury. Concurrently, LBP inhibits NF-κB p65 phosphorylation and nuclear translocation, downregulates proinflammatory factors TNF-α and IL-1β, and consequently suppresses osteoclast activation and bone resorption processes, an effect closely associated with regulation of the NF-κB signaling pathway (114, 115). *In vitro* experiments demonstrated that LBP promotes proliferation and differentiation of MC3T3-E1 cells, enhances ALP activity, facilitates bone mineralization nodule formation, and upregulates RUNX2 and OCN expression. Its mechanism involves activating the ERK1/2 pathway, promoting Nrf2 nuclear translocation, and inhibiting p38, JNK pathway activity, and NF-κB pathway activity (113, 116).

### 3.4 Alkaloid components

Alkaloid components are nitrogen-containing organic compounds with significant physiological activity in traditional Chinese medicine. Some alkaloids exert anti-OP effects by regulating the Nrf2/NF-κB/MAPK pathway.

#### 3.4.1 Berberine

BBR is the main alkaloid component in TCMs such as Coptis chinensis, Phellodendron amurense, and Polygonum cuspidatum, with effects of clearing heat, drying dampness, purging fire, and detoxifying (117). Berberine has undergone small-scale clinical trials for OP and has been shown to effectively improve bone density in postmenopausal patients, reduce bone turnover markers, and modulate gut microbiota, demonstrating strong potential for clinical translation (118, 119). Studies indicate that berberine exerts anti-osteoporosis effects by regulating the Nrf2/NF-κB/MAPK pathway. In an OVX mouse model, oral administration of BBR at doses of 50, 100, and 200 mg/kg for 8 weeks significantly increased BMD, markedly improved trabecular microarchitecture, reduced MDA levels, and downregulated TNF-α expression (120). *In vitro* studies revealed that BBR promotes MC3T3-E1 cell differentiation, enhances ALP activity, facilitates mineralized nodule formation, and upregulates RUNX2 and OCN expression (121). Furthermore, berberine indirectly regulates bone metabolism-related signaling pathways by modulating gut microbiota composition and promoting short-chain fatty acid production, thereby synergistically exerting anti-OP effects (122).

#### 3.4.2 Matrine

Matrine, the main alkaloid component of Sophora flavescens, has properties of clearing heat, drying dampness, killing parasites, and promoting diuresis (123). Currently, studies on matrine are limited to *in vitro* cell and animal experiments, and no clinical trials related to OP have been reported. Research indicates that matrine activates the Nrf2 pathway, suppresses oxidative stress, and promotes osteoblast differentiation. In a hydrogen peroxide-induced osteoblast injury model, matrine intervention significantly increased cell survival rates, markedly reduced ROS and MDA levels, and substantially elevated SOD and GSH activity. The mRNA and protein expression of Nrf2 and HO-1 were significantly upregulated, and Nrf2 nuclear translocation capacity was markedly enhanced (124, 125). Concurrently, matrine significantly inhibited osteoblast apoptosis by upregulating Bcl-2 expression, downregulating Bax and caspase-3 expression, and increasing RUNX2 and OCN expression (126). *In vivo* experiments demonstrated that after matrine intervention in OVX mice, femoral BMD was significantly increased, while Nrf2 and HO-1 expression was upregulated in bone tissue. Concurrently, the phosphorylation levels of NF-κB p65, p38, and JNK were downregulated (127).

#### 3.4.3 Tetrandrine

Tetrandrine is a dibenzylisoquinoline alkaloid derived from tetrandra, a plant of the Menispermaceae family, and exhibits anti-inflammatory, antioxidant, immunomodulatory, and osteoprotective activities (128). Tetrandrine has been investigated in clinical studies related to OP, mainly as a combination therapy to alleviate inflammatory adverse reactions caused by Western medications, and has shown certain adjunctive efficacy in improving bone density and relieving bone pain (129). It broadly regulates the Nrf2/NF-κB/MAPK pathways: by activating the Nrf2 pathway, it upregulates HO-1 and SOD expression, scavenges ROS, alleviates oxidative stress, and protects osteoblast function (130); it simultaneously inhibits the NF-κB pathway by blocking IκBα phosphorylation, suppressing p65 nuclear translocation, downregulating RANKL and inflammatory factors, and inhibiting osteoclast differentiation; it also selectively inhibits excessive activation of p38 and JNK, reduces inflammatory factor release, and alleviates the inflammatory microenvironment, while exerting a mild effect on the ERK1/2 pathway and maintaining osteogenic activity (131). *In vitro* experiments have confirmed that tetrandrine can inhibit RANKL-induced osteoclast differentiation, reduce bone resorption activity, and protect against cytokine-induced osteoblast damage while maintaining osteogenic function (132). *In vivo* studies indicate that tetrandrine improves bone density in OVX mice, reduces trabecular bone destruction, and lowers bone turnover levels, making it an alkaloid monomer with development potential for OP treatment (131).

A comprehensive comparison of the action characteristics of various Chinese herbal monomers shows clear differences in the focus, intensity, and features of pathway regulation among different categories of active components. Flavonoids generally exert mild effects, with favorable antioxidant and anti-inflammatory properties and high safety, making them more suitable for the conditioning of mild OP and long-term daily intervention. Terpenoids show stronger regulatory effects on signaling pathways, especially prominent inhibition of the NF-κB inflammatory pathway, resulting in more evident anti-inflammatory and anti-resorptive effects; however, some terpenoids tend to act on relatively concentrated targets. Alkaloids exhibit strong pharmacological activities with pronounced regulatory effects on bone metabolism, but most are associated with certain pharmacological toxicity, requiring stricter control of clinical dosages. Regarding pathway preferences, most natural components can activate the Nrf2 antioxidant pathway to achieve osteoprotection, whereas their regulation of MAPK sub-pathways differs. Most of these components mainly inhibit the harmful p38 and JNK pathways, whereas activation of the ERK-mediated osteogenic pathway varies among individual compounds. Mechanisms of TCM on OP was listed in **Table 1**.

**Table 1:** Mechanisms and experimental evidence of different categories of TCM regulating the Nrf2/NF-κB/MAPK pathway to intervene in OP.

Category	Monomeric components of TCM	Core mechanism	Key effects	Reference
Flavonoids​	Total Flavonoids from Dipsacus asper (TFD)	↑Nrf2; ↓NF-κB/MAPK	Improves bone structure, anti-ferroptosis	20, 59, 60 61, 62
	Quercetin	↑Nrf2/HO-1; ↓NF-κB/p38/JNK	Reduces osteoclasts; protects osteoblasts	63–67
	Icariin	↑Nrf2/ERK; ↓NF-κB/p38/JNK	↑BMD, anti-oxidative/inflammatory, promotes osteogenesis	37, 69–71, 73, 74
	Medicarpin	↑Nrf2; ↓NF-κB/p38/JNK	Preserves ERK, inhibits osteoclasts	75–77
	Kaempferol	↑Nrf2; ↓NF-κB/p38/JNK	Enhances osteogenesis, anti-inflammatory	60, 78–80, 82
Tercanoids​	Oleanolic acid	↑Nrf2; ↓NF-κB/p38/JNK	Anti-inflammatory, dual bone regulation	84–89
	Dioscin	↑Nrf2/ERK;↓NF-κB/p38/JNK	Improves bone quality, anti-oxidative	72, 90–94
	Ginsenoside Rg1	↑Nrf2/ERK; ↓p38/JNK	Delays bone loss, regulates stem cell fate	37, 89, 96, 98
	Dihydrotanshinone I, Tanshinone I	↑Nrf2; ↓NF-κB/p38/JNK	Protects osteoblasts, anti-resorption	87, 99–101
	Andrographolide	↑Nrf2; ↓NF-κB/p38/JNK	Anti-inflammatory, maintains homeostasis	101, 103–105
Polysaccharides​	APS	↑Nrf2/ERK; ↓NF-κB/p38/JNK	Promotes osteogenesis, anti-apoptosis	107–110
	CDP	↓NF-κB/MAPK	Inhibits osteoclasts, enhances antioxidation	111, 112
	LBP	↑Nrf2/ERK; ↓NF-κB/p38/JNK	Improves bone mechanics, anti-oxidative	37, 113, 114, 116
Alkaloids​	Berberine	Modulates Nrf2/NF-κB/MAPK	Improves BMD, gut microbiota regulation	117–122
	Matrine	↑Nrf2; ↓NF-κB/p38/JNK	Anti-oxidative, protects osteoblasts	123–127
	Tetrandrine	↑Nrf2; ↓NF-κB/p38/JNK	Anti-inflammatory, preserves osteogenesis	128–132

TFD, Total Flavonoids of Drynaria fortunei; APS, Astragalus Polysaccharide; LBP, Lycium barbarum Polysaccharide; CDP, Cistanche deserticola Polysaccharide; ↑, activation/upregulation; ↓, inhibition/downregulation.

## 4 Limitations and outlook

Although progress has been achieved in studies on TCM intervention in OP by targeting the Nrf2/NF-κB/MAPK pathways, the overall research remains dominated by *in vitro* cell experiments and animal model studies. Relevant clinical investigations are limited, and standardized clinical trials with large sample sizes, multicenter designs, and long-term follow-up are still clearly insufficient. This leads to weak clinical evidence, making it difficult to translate basic experimental results into practical clinical diagnosis and treatment. Therefore, the clinical guiding value and relevance of current research still need to be strengthened. Existing reviews in this field mostly focus on listing the pharmacological effects of individual monomers separately and tend to remain descriptive. Systematic horizontal comparison, induction, and integration of the mechanisms of action, efficacy characteristics, and applicable scenarios of different types of TCM monomers are still lacking. These reviews fail to clearly distinguish the common features of similar components and the differences among different categories, making it difficult to establish a unified understanding of their action patterns. Moreover, they cannot provide clear theoretical references for the clinical screening of effective components and rational combination medication, indicating that comprehensive evaluation and deeper inductive discussion remain insufficient. In addition, most current pharmacological regulatory studies still have a partial understanding of MAPK pathway regulation. Most studies only emphasize the inhibitory effects on sub-pathways such as p38 and JNK, but fail to fully recognize the bidirectional regulatory effects of the p38 MAPK pathway, which depend on the intensity and duration of activation. They also overlook the practical issue that moderate activation of this pathway under physiological conditions is beneficial for maintaining normal bone formation, whereas excessive inhibition may disrupt the intrinsic physiological signals of bone metabolism. Consideration of the dynamic balance between moderate activation and excessive inhibition of this pathway is lacking in discussions of intervention strategies using Chinese herbal monomers, resulting in incomplete and imprecise regulatory approaches. First, research on pathway mechanisms is not sufficiently in-depth. Most studies focus on a single pathway or molecule, with weak analysis of pathway crosstalk and regulation of core nodes. Moreover, the use of a single model creates a disconnect from the clinical pathological microenvironment. Second, the correspondence between active components and targets remains unclear. Direct validation experiments between components and key pathway targets are lacking, and research on synergistic mechanisms remains insufficient, which restricts new drug development. Third, the research models are separated from clinical practice. *In vitro* experiments lack simulation of cell interactions, and animal models fail to reproduce the complex clinical etiologies and pathogeneses. In addition, intervention doses differ greatly from clinical applications, and long-term toxicity studies are lacking. Fourth, the quality of clinical studies remains suboptimal, with problems such as small sample sizes, imperfect study designs, and single efficacy evaluation indicators. Research on the relationship between syndrome differentiation and pathway regulation is also insufficient. Fifth, translational research remains weak. The basic-to-clinical translation chain is incomplete, and targeted dosage form optimization and integrated translational research combining traditional Chinese and Western medicine are lacking. Future research should be advanced in six key areas: First, multi-omics and bioinformatics technologies should be used to decipher pathway crosstalk networks. Organoid models should be integrated to elucidate core regulatory mechanisms and clarify component-target-pathway relationships. Second, research models should be optimized by establishing animal models that reflect complex etiologies and pathomechanisms, and *in vitro* models, such as 3D bioprinting, should be used to conduct dose-dependent and long-term toxicity studies. Third, large-scale, multicenter randomized controlled trials should be conducted to expand efficacy evaluation systems, integrate pathway biomarker detection, and establish personalized treatment strategies. Fourth, compound formulations should be optimized based on network pharmacology, novel targeted delivery systems should be developed, and studies on single-component structural modification and integrated Chinese-Western medicine approaches should be performed. Fifth, an integrated “basic-clinical-translational” system should be established to strengthen multidisciplinary collaboration and international partnerships. Sixth, associations among genotypes, gut microbiota, and pathway activity should be explored through precision medicine, thereby expanding pathway regulation studies on OP complications. Based on existing research data, future studies can further conduct cross-sectional comparative and integrative analyses of multiple components to summarize the action patterns of different Chinese herbal monomers in targeted pathway regulation. These monomers can also be classified and organized according to efficacy strength, safety profiles, and functional advantages, thereby overcoming the limitations of descriptive research on single components and improving the systematicity and guiding value of research in this field.

## 5 Conclusion

TCM monomers, such as flavonoids, terpenoids, polysaccharides, and alkaloids, coordinately regulate three core pathological processes, namely oxidative stress, inflammatory response, and apoptosis/differentiation, by targeting the Nrf2/NF-κB/MAPK signaling network. This holistic and multi-targeted approach restores bone metabolic homeostasis. Specifically, these compounds display systemic, bidirectional, or multidirectional regulatory mechanisms. They simultaneously activate the Nrf2 pathway to enhance antioxidant defense, inhibit ferroptosis, and promote osteogenesis, while suppressing NF-κB and p38/JNK activation to alleviate inflammation and inhibit osteoclast differentiation. This “multiple-target” feature directly addresses the complex pathological network of intertwined oxidative stress and chronic inflammation in OP, overcoming the limitations of single-target therapies in traditional Western medicine. The Nrf2, NF-κB, and MAPK pathways participate in close crosstalk, and TCM monoconstituents exert regulatory effects at critical nodes within this network. Activation of Nrf2 not only scavenges ROS but also indirectly inhibits NF-κB activation; suppression of p38/JNK reduces inflammatory cytokine production while alleviating its inhibitory effect on osteoblasts. Through this network-based regulation, TCM monomers disrupt the vicious cycle of “oxidative stress-chronic inflammation-bone metabolic disorder,” reflecting the therapeutic concept of treating both symptoms and root causes. Compounds with diverse structural types, such as quercetin, icariin, oleanolic acid, ginsenoside Rg1, astragalus polysaccharides, and lycium polysaccharides, target this core network through different mechanisms, providing a material basis for developing new compound formulations based on synergistic combinations of effective monomers. In addition, certain components can synergistically regulate bone metabolism through indirect pathways, such as modulation of gut microbiota, further expanding the multidimensional scope of TCM monomers in OP intervention. In summary, TCM monomers restore bone metabolic balance through multi-target regulation by precisely modulating the interactive dialogue within the Nrf2/NF-κB/MAPK signaling network. This not only provides important evidence for clarifying the modern scientific basis of TCM in preventing and treating OP but also lays an important foundation for developing a new generation of anti-osteoporosis drugs with multi-target synergistic advantages and systemic regulatory characteristics. Against the clinical background of the widespread use of modern targeted Western medicines, fully utilizing the advantages of personalized syndrome differentiation treatment in TCM, addressing shortcomings in clinical research, and promoting the translation of basic experimental findings into clinical practice can effectively improve the practical value of Chinese herbal monomers in the clinical prevention and treatment of OP. With the integration of multidisciplinary technologies and continued research, the mechanisms underlying targeted TCM intervention of the Nrf2/NF-κB/MAPK pathway in OP will gradually become clearer. This will provide theoretical foundations and practical references for new drug development, clinical application, and integrated Chinese-Western medicine approaches in TCM-based OP prevention and treatment, ultimately offering more effective therapeutic solutions for the management of this condition.
